# Assessment of a Screening Questionnaire to Identify Exposure to Lead in Pregnant Women

**DOI:** 10.3390/ijerph17249220

**Published:** 2020-12-10

**Authors:** Eléna Coiplet, Marine Freuchet, Claire Sunyach, Julien Mancini, Jeanne Perrin, Blandine Courbiere, Hélène Heckenroth, Christel Pissier, Naima Hamdaoui, Florence Bretelle

**Affiliations:** 1 Department of Gynecology-Obstetric and Reproductive Medicine, Women-Parents-Children Centre (Pôle Femmes-Parents-Enfants/Hôpital La Conception), 147 Boulevard Baille, 13005 Marseille, France; marinefreuchet@hotmail.fr (M.F.); c.sunyach@gmail.com (C.S.); jeanne.perrin@ap-hm.fr (J.P.); blandine.courbiere@ap-hm.fr (B.C.); helene.heckenroth@ap-hm.fr (H.H.); hamdaouinaima2611@gmail.com (N.H.); 2 Centre Hospitalier d’Aubagne, 13400 Aubagne, France; 3 Department of Public Health, Aix-Marseille Univ, APHM, INSERM, IRD, SESSTIM, Hop Timone, BIOSTIC, (Biostatistiques et Technologies de L’information et de la Communication), 13005 Marseille, France; julien.mancini@ap-hm.fr; 4 Aix Marseille Univ, Avignon Université, CNRS, IRD, IMBE, 13005 Marseille, France; 5 Pharmacokinetics and Toxicology Laboratory (Laboratoire de Pharmacocinétique et Toxicologie), AP-HM, 13005 Marseille, France; christel.pissier@ap-hm.fr; 6 Conseil Départemental des Bouches du Rhône—CD13, 13002 Marseille, France; 7 Microbes Evolution Phylogeny and Infections (Microbes Evolution Phylogénie et Infections, MEPHI) IHU—Méditerranée Infection, 19-21 Boulevard Jean Moulin, 13005 Marseille, France

**Keywords:** lead exposure, pregnancy, risk factors, environment

## Abstract

Lead readily crosses the placenta and displays adverse effects on birth outcomes and neurodevelopment. Systematic identification of the risk of exposure during pregnancy is essential but rarely performed, probably due to hospital staff’s workload and their lack of awareness. We aimed to evaluate the relevance of a questionnaire to screen pregnant women for lead exposure. A cross-sectional, multicentre study was carried out on a population of 792 pregnant women from February 2018 to May 2020. A total of 596 women had a blood lead test: 68.5% had blood lead levels below 10 μg/L. The estimated prevalence above 25 µg/L was 4% (95% confidence interval (CI) [2.6–5.9]) and 1.3% had levels above 50 µg/L (95% CI [0.6–2.6]). Multivariate analysis showed that three risk factors significantly increased the probability of blood lead levels above 25 µg/L: the use of traditional cosmetics (adjusted odds ratio [aOR]: 3.90; 95% CI [1.65–9.21]; *p* = 0.002), degraded old housing (aOR: 2.67; 95% CI [1.19–6.038]; *p* = 0.018), and (marginally) eating bread more than twice a day (aOR: 2.40; 95% CI [0.96–6.11]; *p* = 0.060). Our study reveals that a three-question tool can be used to quickly screen for the risk of lead exposure in our population and to trigger lead blood tests and special vigilance during pregnancy follow-up.

## 1. Introduction

Lead has been intensively used since antiquity, causing high dispersion in the environment. Long considered an occupational disease in France, lead poisoning was only recognised in the 1980s as a public health issue with the discovery of several cases of severely poisoned children. From the 1990s onwards, preventive measures taken by public authorities contributed to a sharp reduction in the prevalence of lead poisoning. Nevertheless, the question of the toxic effects of lead on pregnant women and their children remains. In France, major sources of exposure are housing, tap water and industrial pollution. White lead was used in buildings until the middle of the 20th century [1]. Other sources are occupational (industry, public works) and recreational activities (pottery, hunting, fishing). Poisoning can also arise from one’s diet, smoking, traditional medicines and cosmetics (Kohl), and cookware (handcrafted ceramics, tagine dishes). Massive lead poisoning has become rare. However, exposure to low doses is not without consequences. Lead has no known physiological role in humans, and no ‘non-toxic’ threshold could be defined. In France, the High Council of Public Health (HCSP) has recommended intervention for lead concentrations ≥50 µg/L and vigilance for ≥25 µg/L since 2017 [2]. The toxic effects of lead, even at low doses, justify keeping blood lead levels in pregnant women as low as possible. In 2011, less than 1% of women exceeded the threshold of 100 µg/L [3].

Health professionals can rely on guidelines (updated in 2017) to manage lead exposure during pregnancy based on a screening questionnaire [2,4]. Symptomatology is unspecific; the systematic identification of the risk of exposure is therefore essential. Diagnosis relies on blood lead level measurement. Hormonal and calcium metabolism modifications during pregnancy cause a release into blood from bone reserves, increasing the biologically active lead pool [5,6]. Lead readily crosses the placental barrier and is fetotoxic [7]. Studies have shown that prenatal lead exposure increases the risk of abortion or premature delivery, intrauterine growth retardation, low birth weight, high blood pressure and cognitive impairment in children [8,9,10,11,12,13,14,15,16,17,18]. Data during pregnancy remain limited, and prenatal lead exposure from cord blood samples has been analysed [19,20,21]. In our practice, blood lead tests are rarely prescribed during pregnancy follow-up. Time-starved obstetricians must dedicate consultation to medical aspects at the expense of environmental risk factors. To the best of our knowledge, no study has evaluated the use of a screening questionnaire to screen the risk of lead exposure during pregnancy. Thus, we assessed the relevance of the HCSP questionnaire compared to blood testing to save practitioners time. The main objective of our study was to determine the relevance of such a questionnaire to quickly identify lead exposure in pregnant women. The secondary objectives were to estimate the current prevalence of high blood lead levels (≥50 µg/L) in the Marseille urban area and the association with potential obstetric complications associated with lead poisoning.

## 2. Materials and Methods

### 2.1. Study Design

A multicentre, cross-sectional study was carried out from 1 February 2018 to 31 May 2020 on a population of pregnant women, who were followed in the maternity wards of university hospitals in Marseille in a mother infant care centre (MICC) and in a gynaecologist’s private practice in Marseille. A standardised lead exposure screening questionnaire consisting of 18 questions was completed by pregnant women during their first follow-up visit with a gynaecologist or a midwife (Appendix A). The risk factors investigated were a personal or family history of lead poisoning, birth in a country with high lead use (African, Middle Eastern, Southeast Asian, Central American and Eastern European nations), living in dilapidated old dwellings or in proximity to an industrial site, recent renovation work in one’s home, the use of traditional products (medicines, cosmetics, cookware), pica behaviour, the consumption of tap water, alcohol and vegetables, exposure to tobacco, and engaging in a high-risk leisure activity or occupation. A list of occupational and recreational activities was joined to the questionnaire. Our questionnaire was based on the HCSP [2] except for 2 questions regarding diet: eating shellfish and crustaceans, and the consumption of bread more than twice a day, based on the results of ‘L’Étude de l’Alimentation Totale Française 2’ [22] and ‘L’Étude de Santé sur l’Environnement, la Biosurveillance, l’Activité Physique et la Nutrition’ (ESTEBAN) [23], which reported that bread, vegetables, or crustaceans and molluscs are major contributors to lead exposure [20,21,24]. A blood lead level was prescribed systematically. The assay was performed at the hospital sampling centre or in a private laboratory at the patient’s convenience. When the blood lead level was ≥25 µg/L, a control was realised three months later. When the blood lead level was ≥50 µg/L, the lead level was determined on cord blood at delivery, and an environmental survey at the family home was conducted.

### 2.2. Inclusion Criteria

Pregnant women over 18 years old, regardless of their term, who signed informed consent were recruited. They were provided with an information notice.

### 2.3. Data Collection

Questionnaire and lead level results were collected weekly. When the blood lead level was not recorded in the medical file, patients were contacted by telephone. Obstetric history and course of pregnancy information were retrieved from electronic patient records. All data were anonymised and entered into Excel files.

### 2.4. Determining Blood Lead Levels

Blood samples were collected in vacutainer EDTA K2 tubes (Becton Dickinson and Company, Franklin Lakes, NJ, USA). Calibration was based on the standard addition method. One hundred microlitres of whole blood samples were introduced into 15 mL polypropylene tubes along with 100 µL of whole blood provided by the French blood agency (Etablissement Français du Sang; EFS, Marseille, France) and 10 mL of nitric acid 0.5% containing the internal standard (10 µg/L bismuth). The samples were handled using an SPS 4 autosampler (Agilent Technologies, Santa Clara, CA, USA). The mass spectrometer was a Determination by mass spectrometry coupled to an induced plasma (ICP-MS 7800; Agilent Technologies). The operating conditions were checked daily using a 10 µg/L tuning solution (Agilent Technologies). Linearity was validated in the 20–500 µg/L range. Data were processed with Mass Hunter software 4.4 (Agilent Technologies). The method was validated according to European Medicines Agency’s guidelines and accredited by the French committee of accreditation (COFRAC).

### 2.5. Statistical Analyses

The blood lead level was described by its mean (± standard deviation). The prevalence of high blood lead levels (≥25 µg/L and ≥50 µg/L) was estimated with the calculation of an exact 95% confidence interval. The relationship between blood lead levels ≥25 µg/L and each response to the screening questionnaire was analysed with the Wald test from univariable binary logistic regression models. The same test was employed to study the relationship between blood lead levels ≥25 µg/L and obstetrical history. Student’s *t*-test was used for mean comparisons. A multivariate binary logistic regression analysis was then run to study the variables independently associated with blood lead levels ≥25 µg/L. Only the variables significant in univariate analysis were entered in this model. Additional stepwise analyses did not identify any other significant variables. Receiver Operating Characteristic (ROC) curves were used to estimate the ability of different scoring methods (using all the questions or only those selected in the logistic regression) to distinguish women with blood lead levels ≥25 µg/L using the area under the curve (AUC). All analyses were performed using IBM SPSS Statistics 20.0 software (IBM Inc., New York, NY, USA). A value of *p* ≤ 0.05 was considered statistically significant.

### 2.6. Authorizations

This study has been declared to the ‘Commission de l’Informatique et des Libertés’ (CIL) (2018-13) and was approved by the local ethics committee (2018-27-09-004 and 2018-18-10-005). Informed consent was obtained from the participants.

## 3. Results

During the inclusion time, 11% of the women consulting for pregnancy in the hospital ward completed the questionnaire. Patients in our sample had their pregnancy follow-up performed in university hospitals (93.8%), in private practice (5.4%) and in an MICC (5.4%). The screening questionnaire was completed by 792 patients; 596 (75.3%) women had their blood lead levels measured. The country of birth was France for 58.3% of the women. Among immigrant women, 41.5% were born in a country with high lead use, mainly Algeria (13.2%) and Comoros (7.5%). Maternal age was 30.46 ± 5.88 years. Blood tests were performed in the course of the 1st, 2nd or 3rd trimester for 46.9%, 21.7% and 31.4% of the patients, respectively.

**Figure 1:**
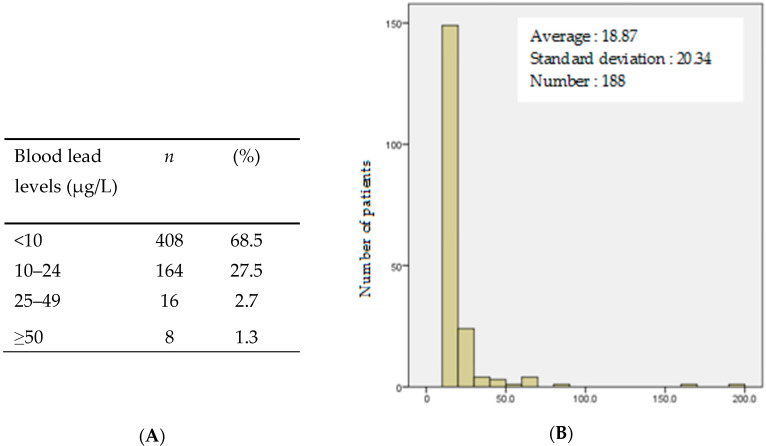
Distribution of blood lead levels in the sample (**A**) Presents categorical distribution of blood lead level values (*n* = 596). Note that laboratories sometimes reported “below 10 µg/L” rather than accurate value. (**B**) Distribution of blood lead levels above 10 µg/L.

The prevalence of blood lead levels above the 25 µg/L HCSP vigilance threshold and above the 50 µg/L intervention threshold was 4% (95% confidence interval (CI) [2.6–5.9], *n* = 25) and 1.3% (95% CI [0.6–2.6], *n* = 8), respectively. Seven patients (0.9%) reported no risk factors for lead exposure, and 3 of them had a blood lead level below 10 µg/L. The other four did not do the test. Out of 596 samples taken, 408 (68.5%) revealed lead levels strictly below 10 µg/L, and 188 (31.5%) women had lead levels above 10 µg/L, with an average of 18.8 ± 20.3 µg/L (Figure 1). The risk factors for lead exposure, according to their frequency, are summarised in Table 1. The most common sources of exposure were drinking tap water (75.5%), the consumption of bread more than twice a day (51.3%), and birth in a country with high lead use (43.4%). The 596 lead tests and the analysis of the questionnaires allowed us to identify risk factors associated with lead poisoning (Table 2). The risk of a high blood lead level (≥25 µg/L) was significantly increased by the use of traditional cosmetics (*p* = 0.001), degraded old housing (*p* = 0.019), and the consumption of bread more than twice a day (*p* = 0.045). However, the results were at the limit of significance for birth in a country with high lead use (*p* = 0.076) and living close to an industrial site (*p* = 0.102). Despite being not statistically significant, other factors were rather strongly associated with lead poisoning (odds ratio >2): living close to an industrial site, a high-risk country of birth, and history of lead poisoning (Table 3).

Women poisoned reported an average of 4.9 ± 1.8 potential sources of lead exposure, which was significantly higher than in the blood lead level group < 25 µg/L (4.9 ± 1.8 vs. 3.9 ± 1.9; *p* = 0.009). Univariate analysis did not show a significant association of clinical characteristics according to the level of lead impregnation (Table 4). At the end of inclusion, 52.5% of patients had given birth; 83.6% gave birth after 37 weeks of gestation (WG), with an average of 38.6 ± 3.3 WG. The gender distribution was equal (199 girls and 207 boys), and the average birth weight was 3077 ± 739 g. There was no significant difference between the 2 groups in the risk of intrauterine growth restriction (IUGR) (*p* = 0.177), anaemia (*p* = 0.701), preterm delivery (*p* = 1.0), hypertension (*p* = 1.0) or birth weight (*p* = 1.0) (Table 4).

**Figure 2:**
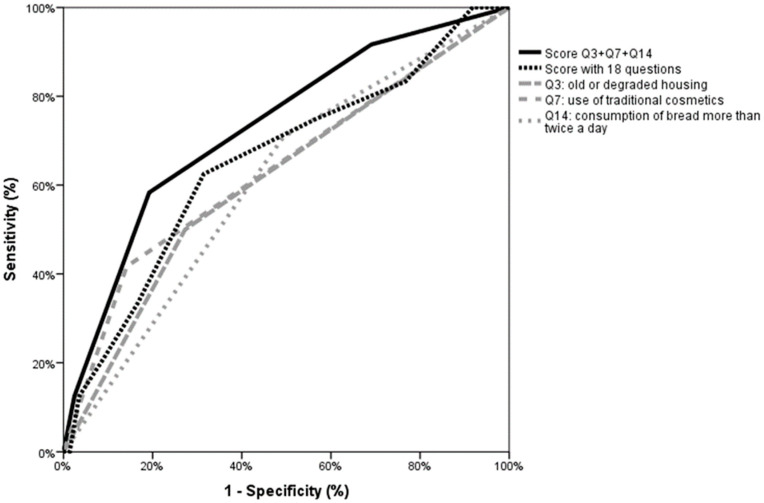
Comparison of ROC curves considering Q3, Q7, Q14 individually, Q3 + Q7 + Q14 or all 18 questions. When all 18 questions were considered, the area under the curve was 0.654 (95% confidence interval (CI) [0.538–0.771], *p* = 0.010). When only the three significant questions were taken into account the area under the curve is 0.731 (95% CI [0.626–0.836], *p* < 0.001). Individual areas under the curve (AUCs) for those teach three questions are 0.614 (95% CI [0.493–0.734]) for Q3, 0.638 (95% CI [0.511–0.764]) for Q7, and 0.608 (95% CI [0.497–0.718]) for Q14.

According to the multivariate analysis, the three risk factors identified in univariate analysis independently increased the risk of blood lead levels above 25 µg/L (Table 3). Use of traditional cosmetics (OR: 3.90; 95% CI [1.65–9.21]; *p* = 0.002), living in degraded old housing (odds ratio (OR): 0.67; 95% CI [1.19–6.038]; *p* = 0.018), and the consumption of bread more than twice a day (OR: 2.40; 95% CI [0.96–6.11]; *p* = 0.060). The ROC curve shows that considering only the three risk factors significantly associated with high blood lead levels tended to be more discriminating for the detection of a high blood lead level than summing up the 18 questions (Figure 2). When patients mentioned only one of the three questions, the risk reached 2.7%, which increased to 10.3% with two positive questions and to 17.6% with three positive questions (Table 5).

Eight women had a blood lead level ≥50 µg/L. The four newborns tested had a blood lead level above 25 µg/L, with an average of 95.5 µg/L (55 to 194 µg/L). Their average age was 38 (27 to 46). The patients mainly originated from North Africa (one from Morocco, five from Algeria). The highest level of lead impregnation tested was 194 µg/L in a 30-year-old Algerian-born woman who arrived in France 3 months before her first visit to the maternity yard. She had completed three pregnancies without particularities and was 12 WG. She reported 5 risk factors for lead exposure: country of origin, old habitat, use of Kohl and the consumption of tap water and bread. The pregnancy was marked by gestational diabetes mellitus. She gave birth to a full-term eutrophic boy. The cord blood lead level was 264 µg/L. The environmental survey at home revealed peeling paint and the use of a stove imported from Algeria.

## 4. Discussion

Reducing child lead poisoning and preventing impacts on health during infancy and adulthood involves identifying pregnant women at risk. As clinical signs are not specific, only a strong action to investigate exposure risk factors, followed by the prescription of a blood test if factors are identified, can detect poisoned patients. However, in France, this is not systematically performed during pregnancy, probably due to hospital staff’s workload and their lack of awareness. Our study shows that three questions are enough to screen for risk of lead exposure in our population. This is particularly important since maternity wards of university hospitals and the MICC in southern France provide pregnancy care for pregnant women born in countries with a high level of lead use and/or living in precarious housing conditions, a population potentially at risk of lead poisoning.

### 4.1. Towards an Efficient and Simple Screening Lead Poisoning Questionnaire

The French HCSP proposed an 18-item questionnaire and recommended prescribing a blood lead test if the answer to at least one question indicated possible lead exposure. Almost all women tested reported at least one risk factor. According to the HCSP recommendations, blood lead levels should be prescribed to almost all pregnant women. This can be discussed with regard to the workload of health professionals and to health costs. We found that only 3 of the 408 women with blood lead levels below 10 µg/L reported no potential lead exposure factor. This suggests that the questionnaire proposed by the HCSP cannot fully distinguish women reporting one lead exposure risk factor from those with lead poisoning attested by high blood lead levels. On the other hand, women with blood lead levels >25 µg/L reported 4.9 ± 1.8 potential lead exposure sources. Hence, lead poisoning appears complex and cannot be traced to a single source. Almost all our patients reported at least one risk factor for lead exposure in the questionnaire; however, the analysis revealed that the use of traditional cosmetics, degraded housing and the consumption of bread more than twice a day significantly increased the risk of high blood lead levels. A history of lead poisoning showed a high odds ratio, but due to the rarity of the event, this risk was not significant. Tobacco smoking, alcohol consumption, age and old housing were the chief factors associated with increased blood lead levels reported in the ESTEBAN study performed among the general population [23]. By contrast, it reported no association between elevated blood lead levels and the use of traditional cosmetics.

### 4.2. A snapshot of Lead Poisoning in a Pregnant Population Consulted at University Hospital in Marseille

In our population, almost all women (95.1%) lived in an area of Marseille where 32% of the housing units were built before 1949. We found a prevalence of lead poisoning ≥50 µg/L of 1.3% (95% CI [0.6–2.6]), which is slightly higher than in recent studies [25]. Differences in biological matrices (venous blood vs. umbilical cord blood) could account for these results. Importantly, we confirmed a significant decrease in lead exposure as previously stated [24,26]. The mean blood lead level above 10 µg/L was 18.8 µg/L, as reported before [23]. The HCSP’s 2017 updated guidelines do not recommend particular pregnancy follow-up when blood lead levels are below 50 µg/L. Even if we did not find any link between elevated blood lead levels and obstetric complications, unlike recent studies [18,27,28], we recommend controlling the blood lead level when the first test is above 25 µg/L. Efforts should be made to reduce lead exposure in these patients.

### 4.3. Implications for Health Professionals and Patients

Lead poisoning is rare but probably underdiagnosed due to heterogeneous screening strategies. Health professionals should inform patients about lead poisoning and promote the screening process. The under-diagnosis of lead exposure is probably worse in our population. In our sample, 41.5% of the women came from high-risk countries. If all patients were prescribed a lead test, 196 (24.7%) did not take it, indicating that the public is likely poorly informed about lead poisoning. In our practice, women also often miss their pregnancy follow-up visits. This could be explained by precarious social, language and cultural barriers, as well as poor accessibility to means of transport. Even if questionnaires appear to be an easy way to screen for risk factors, obstacles probably remain due to hospital staff’s high workload and lack of awareness. Apart from saving time for practitioners, this 3-question screening could be simpler to understand for our non-French speaking patients. It is easy to carry out for pregnancy follow-up visits and should help to address the crucial public health issue of lead exposure. Our 3-question tool could implement a lead intoxication prevention policy and thereby help reduce health inequalities.

### 4.4. Strengths and Weaknesses of the Study

To our knowledge, we have provided the first evaluation of a lead poisoning screening questionnaire. We are nevertheless aware of a selection bias arising from inclusion in university hospital maternity wards and the MICC in Marseille, which recruit a large number of patients in precarious situations. In addition, the questionnaire and lead blood test were only proposed to a small number of patients (11%). This likely reflects the fact that obstetricians and midwives are poorly informed of lead exposure consequences for pregnancy complications, issues and child health. Consequently, neither inquiring nor blood testing are included in their clinical approach. Even if the population of pregnant women consulting in our hospital is rather socially homogeneous, we cannot rule out selection bias. However, despite the small size of our sample, which underpowered our study, our results are consistent with previously published studies [22].

## 5. Conclusions

The health impact of environmental pollution is a critical public health issue. Our study confirms a decrease in lead poisoning in southern France. However, this remains a concern given the potential effects on the course of pregnancy and child neurodevelopment. We showed that a simple three-question tool can be used to quickly screen for the risk of lead exposure during pregnancy and trigger special vigilance and blood lead tests during follow-up. We strongly believe that our study will allow time-starved clinicians to screen for this environmental toxic exposure and deliver appropriate individual healthcare to lead exposed pregnant women. Importantly, our study could help to achieve the UN’s 2030 Sustainable Development Goals (SDGs) 3 (‘good health and well-being’) and 10 (‘reduced inequalities’) [29].

## Figures and Tables

**Table 1:** Frequency of lead exposure risk factors mentioned in the questionnaire.

	YES Number of Patients (*n*) Frequency (%)	NO Number of Patients (*n*) Frequency (%)	I Don’t Know Number of Patients (*n*) Frequency (%)
Tap water consumption (Q9) ^a^	598 75.5	193 24.4	1 0.1
Bread consumption > 2 times/day (Q14)	406 51.3	384 48.5	2 0.3
Birth in a high-risk country ^b^ (Q2)	344 43.4	448 56.6	0 0
Active or passive smoking (Q12)	310 39.1	486 61.4	28 3.5
Vegetables consumption > 2 times/day (Q15)	305 38.5	470 59.3	17 2.1
Living in an old and deteriorated dwelling (Q3)	217 27.4	571 72.1	4 0.5
Use of traditional containers (tagine dish), handmade ceramic, tin or crystal containers (Q8)	177 22.3	609 76.9	6 0.8
Exercise of a hobby or profession at risk ^c^ (Q18)	155 19.6	634 80.1	3 0.4
Consumption of shellfish and crustaceans (Q16)	152 19.2	639 80.7	1 0.1
Use of traditional cosmetics (kohl) (Q7)	115 14.5	676 85.4	1 0.1
Carrying out recent renovation work in an old home (Q4)	105 13.3	686 86.6	1 0.1
Consumption of traditional medicines or food supplements (Q5)	53 6.7	736 92.9	3 0.4
Living close to an industrial site (Q17)	33 4.2	740 93.4	3 0.4
Alcohol consumption (Q13)	25 3.2	761 96.1	1 0.1
Pica behaviour (Q6)	23 2.9	766 96.7	3 0.4
Personal or family history of lead poisoning (Q1)	7 0.9	778 98.2	7 0.9

^a^ Q refers to questions of the questionnaire (Appendix A). ^b^ Blood lead levels in the general population are often lower in France than in some countries in Africa, the Middle East, from Southeast Asia, the West Indies and Eastern Europe. ^c^ These activities include crafts (print making, stained glass, ceramics), outdoor sports (hunting and fishing), liquor distillation, creating stained glass, enamelling copper, casting bronze, pottery with certain leaded glazes and paints; casting ammunition, jewellery making and electronics (with lead solder), glassblowing with leaded glass.

**Table 2:** Univariate analysis of exposure sources according to the level of lead impregnation (*n* = 596).

	Blood Lead Level		*p* ^a^
	<25 µg/L (*n* = 572)	≥25 µg/L (*n* = 24)	
	*n* (%)	*n* (%)	
History of lead poisoning	5 (0.9)	1 (4.2)	0.153
High-risk country of birth ^b^	250 (43.7)	15 (62.5)	0.076
Degraded old housing	156 (27.3)	12 (50)	0.019
Renovation work in an old dwelling	77 (13.5)	5 (20.8)	0.310
Use of traditional medicines or food supplements	37 (6.5)	2 (8.3)	0.718
Pica behaviour	16 (2.8)	1 (4.2)	0.695
Use of traditional cosmetics	81 (14.2)	10 (41.7)	0.001
Use of traditional food containers (tagine dish), handmade ceramic, tin or crystal containers	123 (21.5)	5 (20.8)	0.938
Tap water consumption	430 (75.2)	18 (75)	0.985
Active or passive smoking	230 (41.6)	8 (34.8)	0.530
Alcohol consumption	20 (3.5)	1 (4.2)	0.862
Consumption of shellfish and crustaceans	103 (18)	5 (20.8)	0.725
Bread consumption >2 times/day	282 (49.3)	17 (70.8)	0.045
Vegetable consumption >2 times/day	218 (38.1)	9 (37.5)	0.952
Living close to an industrial site	14 (2.4)	2 (8.3)	0.102
Exercise of a hobby or profession at risk ^c^	99 (17.3)	2 (8.3)	0.264

^a^*p*-value from Wald test (binary logistic regression model). ^b^ Blood lead levels in the general population are often lower in France than in some countries in Africa, the Middle East, from Southeast Asia, the West Indies and Eastern Europe. ^c^ These activities include crafts (print making, stained glass, ceramics), outdoor sports (hunting and fishing), liquor distillation, creating stained glass, enamelling copper, casting bronze, pottery with certain leaded glazes and paints; casting ammunition, jewellery making and electronics (with lead solder), glassblowing with leaded glass.

**Table 3:** Multivariate analysis of factors significantly associated with lead poisoning.

	Univariable Analysis		Multivariable Analysis		
	Crude Odds Ratio	95% CI	Adjusted Odds Ratio ^a^	95% CI	*p*
Living close to an industrial site	3.62	[0.78–16.92]	NE ^b^		
High risk country of birth ^c^	2.15	[0.92–4.99]	NE		
Consumption of bread more than twice per day	2.50	[1.02–6.11]	2.40	[0.96–5.99]	0.060
Use of traditional cosmetics	4.30	[1.86–10.08]	3.90	[1.65–9.2]	0.002
Degraded old housing	2.67	[1.17–6.06]	2.76	[1.19–6.38]	0.018
History of lead poisoning	4.93	[0.55–43.9]	NE		
Renovation work in an old dwelling	1.70	[0.61–4.66]	NE		
Use of traditional medicines or food supplements	1.31	[0.30–5.81]	NE		
Pica behaviour	1.51	[0.19–11.88]	NE		
Use of traditional food containers (tagine dish), handmade ceramic, tin or crystal containers	0.96	[0.35–2.62]	NE		
Tap water consumption	0.99	[0.38–2.54]	NE		
Active or passive smoking	0.62	[0.18–2.12]	NE		
Alcohol consumption	1.2	[0.15–9.33]	NE		
Consumption of selfish or crustaceans	1.20	[0.43–3.29]	NE		
Vegetable consumption more than twice per day	0.97	[0.42–2.27]	NE		
Exercise of a hobby or profession at risk ^d^	0.43	[0.10–1.87]	NE		

^a^ Each odds ratio is adjusted for the two other variables entered in the model. ^b^ Not Entered in the multivariate binary logistic regression model. ^c^ Blood lead levels in the general population are often lower in France than in some countries in Africa, the Middle East, from Southeast Asia, the West Indies and Eastern Europe. ^d^ These activities include crafts (print making, stained glass, ceramics), outdoor sports (hunting and fishing), liquor distillation, creating stained glass, enamelling copper, casting bronze, pottery with certain leaded glazes and paints; casting ammunition, jewellery making and electronics (with lead solder), glassblowing with leaded glass.

**Table 4:** Univariate analysis of clinical characteristics according to the level of lead impregnation (*n* = 596).

	Blood Lead Level		*p* ^a^
	<25 µg/L (*n* = 572)	≥25 µg/L (*n* = 24)	
	*n* (%)	*n* (%)	
Age			
>35 years old	112 (19.6)	7 (29.2)	0.294
Obstetrical history			
Spontaneous miscarriage	134 (26.4)	5 (21.7)	0.617
Prematurity (<37 WG)	24 (10.2)	1 (4.3)	0.451
IUGR	19 (3.8)	1 (4.3)	0.596
Pregnancy hypertension	22 (4.4)	0 (0.0)	1
Pregnancy course			
Anemia (Hb < 11 g/L)	137 (32.6)	7 (36.1)	0.701
IUGR	28 (7.4)	3 (15.8)	0.177
Pregnancy hypertension	15 (4)	0 (0)	1
Prematurity (<37 WG)	37 (9.8)	1 (5.6)	1
Low birth weight (<2500 g)	48 (14)	2 (14.3)	1

IUGR: Intrauterine Growth Restriction, ^a^ *p*-value from Wald test (binary logistic regression model).

**Table 5:** Calculation of an overall score from exposure factors significantly associated with high blood lead levels (*n* = 596).

	Blood Lead Level ≥25 µg/L	
Score Q3 + Q7 + Q14 ^a^	N	%
0 (*n* = 179)	2	1.1
1 (*n* = 293)	8	2.7
2 (*n* = 107)	11	10.3
3 (*n* = 17)	3	17.6

^a^ Q3, Q7 and Q14 refer to the questions of the questionnaire, i.e., old or degraded housing, use of traditional cosmetics, and consumption of bread more than twice a day. For example, a score of 3 indicates a positive response to all three questions.

## Appendix A. Lead Poisoning Screening Questionnaire

(Based on the questionnaire proposed in the High Council of Public Health (HCSP) guidelines) Haut Conseil de la Santé Publique [2].

Q1: Have you ever had a personal health history with lead poisoning (acute and chronic absorption of lead into the body)? Or has someone in your surroundings?	YES ❑ NO ❑
Q2: Were you born in a country in Africa (including Comoros, Madagascar), the Middle East, Southeast Asia, the West Indies or Eastern Europe?	YES ❑ NO ❑
Q3: Do you live in an old and degraded apartment (built before 1975)?	YES ❑ NO ❑
Q4: Over the last 6 months, have you been in contact with someone working in construction, in an industrial site, or have you been living in an old apartment or in an apartment in construction (spread of dust, including stripping or sanding old paint)?	YES ❑ NO ❑
Q5: Do you ingest traditional food supplements (clays, medicinal herbs?)	YES ❑ NO ❑
Q6: Have you ever eaten non-food substances such as clay, soil, plaster, paint flakes?	YES ❑ NO ❑
Q7: Do you use traditional cosmetics? (kohl, surma)?	YES ❑ NO ❑
Q8: For the preparation/preservation of food, do you often use traditional containers (tajine dishes), enamelled ceramic pottery, pewter, or crystal dishes?	YES ❑ NO ❑
Q9: Do you drink tap water?	YES ❑ NO ❑
Q10: if yes, are there any lead pipes?	YES ❑ NO ❑
Q11: Do you smoke?	YES ❑ NO ❑
Q12 Are you passively exposed to the smoke of cigarettes?	YES ❑ NO ❑
Q13: Have you been drinking alcohol while pregnant?	YES ❑ NO ❑
Q14: Do you eat bread more than twice a day?	YES ❑ NO ❑
Q15: Do you eat vegetables grown close to an industrial site more than twice a day?	YES ❑ NO ❑
Q16: Have you been eating seafood while pregnant?	YES ❑ NO ❑
Q17: Do you live or spend much time in places close to an industrial site releasing lead into the atmosphere?	YES ❑ NO ❑
Q18: Do you practice (or have you practiced) shooting, hunting, fishing, pottery or a risky professional activity (in the industrial sector, construction work, metallurgy, in contact with objects containing lead)? Or is there in your surroundings someone practicing such leisure activities or working in those areas?	YES ❑ NO ❑
